# Female-biased birth sex ratio in a female dispersal primate suggests local resource competition

**DOI:** 10.1098/rsbl.2024.0002

**Published:** 2024-05-01

**Authors:** Carola Borries, Andreas Koenig

**Affiliations:** ^1^ Department of Anthropology and Interdepartmental Doctoral Program in Anthropological Sciences, Stony Brook University, Stony Brook, NY, USA; ^2^ Graduate Program in Ecology and Evolution, Stony Brook University, Stony Brook, NY, USA

**Keywords:** adult sex ratio, kin competition, Phayre’s leaf monkeys, sample size effects, sex-differential mortality, *Trachypithecus phayrei crepusculus*

## Abstract

Group living may entail local resource competition (LRC) which can be reduced if the birth sex ratio (BSR) is biased towards members of the dispersing sex who leave the group and no longer compete locally with kin. In primates, the predicted relationship between dispersal and BSR is generally supported although data for female dispersal species are rare and primarily available from captivity. Here, we present BSR data for Phayre’s leaf monkeys (*Trachypithecus phayrei crepusculus*) at the Phu Khieo Wildlife Sanctuary, Thailand (*N* = 104). In this population, nearly all natal females dispersed, while natal males stayed or formed new groups nearby. The slower reproductive rate in larger groups suggests that food can be a limiting resource. In accordance with LRC, significantly more females than males were born (BSR 0.404 males/all births) thus reducing future competition with kin. This bias was similar in 2-year-olds (no sex-differential mortality). It became stronger in adults, supporting our impression of particularly fierce competition among males. To better evaluate the importance of BSR, more studies should report sex ratios throughout the life span, and more data for female dispersal primates need to be collected, ideally for multiple groups of different sizes and for several years.

## 1. Introduction

The balance and imbalance of animal birth sex ratios (BSRs), also called secondary sex ratio [1], have been a research topic for more than 150 years [2–4], (overviews in [5,6]). It is considered a classic example of natural selection acting on the individual while modifying a population characteristic (i.e. BSR). The first quantitative work [2] and subsequent research [4,7,8] focused on reasons why BSR should be even. It was argued that an uneven BSR would not be evolutionary stable because offspring of the rarer sex would have a reproductive advantage, rendering the production of the rarer sex more rewarding. This, in turn, would push the sex ratio back into balance [9].

However, differential fitness returns based on competition over local resources could make it beneficial for biasing BSR in order to reduce competition with kin [10–13]. When more offspring of the dispersing sex are produced, that is, BSR is biased, then local resource competition (LRC) is lowered [10,14,15]. Individuals of the dispersing sex will leave the area prior to first reproduction, thereby reducing competition with their kin (parents and siblings).

Two additional scenarios related to LRC are distinguished in the literature [16,17]: local mate competition [9,18] and local resource enhancement [19,20]. On the one hand, local mate competition can occur in small, subdivided populations of certain non-mammalian organisms. In such scenarios, a small number of females, often two or three, produce related males who compete over mating access to their sisters. This should result in a female-biased BSR depending on the number of breeders and the extent of sibling mating [12,16,18]. On the other hand, local resource enhancement can occur in highly cooperative species if support from one sex increases the fitness of their relatives, for example, when adult individuals delay breeding to help raise their younger siblings. In this scenario, the BSR should be biased towards the helping sex [19,20]. In non-human primates, this helper-at-the-nest model has been applied successfully to BSR biases in cooperative breeders [21].

Most research on BSR and its biases has thus far been performed in birds [22,23], invertebrates [24] and mammals [15]. Within mammals, the bulk of the data stems from the order Primates [14,25–27] with an emphasis on cercopithecine primates for which most data are available. This uneven sample likely causes an overrepresentation of species with male-biased dispersal [28,29]. More specifically, of the 56 primate species with sufficiently large sample sizes ( ≥ 100 births) included in the BSR analysis of Silk & Brown [21] (detailed below), 66% showed male-biased dispersal, 14% female-biased dispersal and in 20% both sexes dispersed (calculated from fig. 1 in [21]). In accordance with LRC, the authors confirmed a female-biased BSR in species with female-biased dispersal, a male-biased BSR if males were the primary dispersers and an intermediate BSR when both sexes dispersed in similar proportions. However, all eight BSRs from species with female-biased dispersal stem from captive populations [21] (JB Silk 2023, personal communication), where effects of resource limitation and resource competition are minimal and predation risk is absent. Two data points for wild species with female-biased dispersal were excluded because the sample size was below 100 (57 and 59 births, respectively). As shown in their study, BSR based on small sample sizes produced the strongest biases. This effect disappeared when only samples of at least 100 births were considered (see also [30] for a similar sample size effect on the twinning rate in primates). To examine LRC more thoroughly, BSRs for wild primates with female-biased dispersal are needed.

While a biased BSR suggests a specific competitive scenario as outlined above, ultimately the sex ratio of adult individuals matters the most. It is therefore important to trace the sex ratio through time, for different age classes as well as in adults. A classic example is the male-bias in human BSR [7,31,32] which disappears during adulthood and reverses in old age [33]. It has long been assumed that this bias compensates for a higher mortality in male blastocysts, embryos, neonates and immatures (e.g. [34,35]), but it has since been shown that the sex ratio at conception is even, and overall female mortality *in utero* is higher (not lower), leading to a male-biased BSR in humans [31,32]. Similar ratio changes were reported for captive spider monkeys (*Ateles* spp. [36]), for which the female-biased BSR had become male-biased at maturity. In contrast, a male-biased BSR in captive galagoes (*Galago crassicaudatus*) persisted into adulthood [10] and in wild Nepal grey langurs, an almost even BSR was still present in 2-year-olds (*Semnopithecus schistaceus* [37]). Shifts in ratio reflect sex-differential mortality in maturing individuals which likely depends on multiple factors and cannot be predicted based on LRC alone. Consequently, the sex ratios of older age groups, if available, should be included in sex ratio analyses to evaluate the long-term effects of BSR biases.

Here, we provide BSR and mortality data for wild Phayre’s leaf monkeys (*Trachypithecus phayrei crepusculus*). A subsample of our BSR data was already included in Silk & Brown [21] but did not contribute to their main analysis because, at the time, our sample size only was 57 births (cf. above). In Phayre’s leaf monkeys, natal dispersal is female-biased with all but one female dispersing before reaching sexual maturity [38] (C Borries *et al.* 2024, unpublished data). With an average of about seven adult females per group and resident natal males being a mix of half-brothers and unrelated males [39,40], local mate competition is unlikely to apply. In addition, immature and adult females exhibit allomaternal care [40,41]. However, this behaviour differs from helpers-at-the-nest scenarios because most females within a group are unrelated and do not delay breeding, rendering local resource enhancement unlikely.

With respect to LRC at the study site, we previously showed that reproductive rates were slower in larger groups suggesting that individuals compete locally for food [42]. Consequently, because dispersal is female-biased, we predicted BSR to be female-biased as well. To estimate long-term effects, furthermore, we examined if a sex ratio bias persisted in older individuals.

## 2. Methods

The study was conducted at Huai Mai Sot Yai (16°27′ N, 101°38’ E at 600–800 m a.s.l.) within the Phu Khieo Wildlife Sanctuary, Thailand from January 2001 to January 2009 [43]. With few exceptions, four groups were contacted for several consecutive days each month for 3.3–7.6 years each (table 1), amounting to more than 23 000 contact hours. For every day in contact with a group, we recorded all individuals encountered and identified missing or new group members. All members of the focal groups were individually recognized based on natural markers such as fur colour, shape and length of whiskers and crest, the tail length and the shape of depigmented areas around the eyes, the lips and below the belly button [39]. Unlike some primates such as gibbons or gorillas, the sex of Phayre’s leaf monkeys can be determined at birth. In males, the penis is visible and in females, the vulva separates the ischial callosities (or sitting pads). However, visibility in the dense forest habitat was poor and the small newborns were often higher than 25 m up in the trees. Because newborns were constantly clinging to a conspecific, chances for sexing them were best when they were switched between caretakers or when their genitalia were inspected or groomed. We paid particular attention to determine the sex of every newborn within its first days of life which is why only one individual remained unsexed (i.e. < 1% unknown).

**Table 1.** Birth sex ratio (BSR) in the study population by group. A low number of births does not necessarily reflect a small female group size because groups were studied for a varying number of years. The total is found across all groups and years.

group	N births	females	males	BSR	study years
PA	31	17	14	0.452	7.6
PB	36	19	17	0.472	5.4
PO	17	13	4	0.235	3.3
PS	20	13	7	0.350	6.8
**total**	**104**	**62**	**42**	**0.404**	

Following Silk & Brown [21] and most other studies on the topic, we report BSR as the proportion of males in all births. This value can theoretically vary between zero and one. A value above 0.5 indicates a male bias and a value below 0.5 indicates a female bias. To facilitate comparisons with other indices, we also provide the sample sizes for males and females separately.

Survival rates to 1 and 2 years of age, respectively, were determined for those individuals who could have reached the respective age during our study period. We did not consider the cause of mortality because most infants simply disappeared. We could not consider individuals older than 2 years because the youngest natal female was 2.7 years when she emigrated and the youngest natal male was 1.9 years (C Borries *et al*. 2024, unpublished data), although males rarely dispersed and usually at a much older age [39].

Female Phayre’s leaf monkeys were considered adults at first parturition at an average age of 5.3 years (range = 4.8–6.2 years, *n* = 17 [43]). Males were considered adults upon completion of structural growth. To determine growth cessation, all observers independently assessed the sitting height of maturing males visually every month for changes. The interobserver agreement was high. Males were considered adults at a mean age of 5.1 years (range = 4.8–5.4, *n* = 8). This estimate is very similar to the age at growth cessation determined via photogrammetry (mean = 4.8 years, range = 4.5–5.3 years, *n* = 6 [44]).

To determine the adult sex ratio (ASR) in mixed-sex groups, we used two approaches. First, ASR was based on the composition of the focal groups in January of each year from 2004 to 2009, thus avoiding repeated daily or monthly group counts. We only considered years with data for at least three focal groups and excluded the first years of the study before 2004. This led to six yearly assessments including 22 group values. The resulting six yearly ASRs were then averaged. Second, to maximize the number of groups considered, ASR was based on six separate censuses performed between December 2002 and June 2004 of up to six mixed-sex groups per census. While spanning a shorter period, this sample included the focal groups and high-quality counts of three additional neighbouring groups. Phayre’s leaf monkeys are territorial with a small range overlap [45], such that non-focal groups can be identified by location and via some known individuals (see identification of individuals above). The mean was found by averaging ASR for all six censuses. Because both calculations included values for our focal groups, the two approaches are not independent.

We note that both ASRs only refer to group living individuals and do not consider solitary males nor small groups of multiple males, which were encountered on occasion [39]. These ‘all-male-bands’ were formed when multiple males left a group to settle next to their original group. They were later joined by females, resulting in a new mixed-sex group. We never observed male immigration into an already existing group [39]. In contrast, females were rarely encountered on their own. Prior to first parturition, they disappeared or dispersed mainly during group encounters, thus leaving the natal group for another mixed-sex group (C Borries *et al*. 2024, unpublished data).

## 3. Results

Of the 105 infants born into our four study groups, one was unsexed, 42 were males and 62 were females. With a value of 0.404, the BSR was significantly female-biased (binomial test *p* = 0.031, one-tailed). If the one unsexed individual would have been a male, the BSR would have still been significantly female-biased. This unsexed individual is not considered further. In all four study groups, the BSR was female-biased even though the sample size is naturally lower for individual groups (range of 17–36 births, table 1) and would not reach statistical significance. As expected, the strongest skew in BSR was found in the two groups with the lowest number of births (table 1).

The sex ratio of 1-year-olds and 2-year-olds remained female-biased (0.429 and 0.415, respectively, table 2), reflecting the fact that in these age classes, survival rates were similar for males and females. ASR in mixed-sex groups was more strongly biased towards females. For our focal groups, the ASR averaged 0.201 (range = 0.167–0.250). Based on group censuses, we determined a similar ASR (mean = 0.248 and range = 0.185–0.292). Even if extra-group males could have been considered, ASR is likely below 0.300 and the female bias in sex ratio is strongest in adults.

**Table 2.** Birth sex ratio (BSR) for different age classes across all four study groups. For further explanations about the adult sex ratio (ASR) see §2.

	sample size			sex ratio		percent surviving		
age when	*N*	females	males	sex ratio	*p*-value^a^	females	males	*p*-value^b^
newborn	104	62	42	0.404	0.031	n/a	n/a	
1-year-old	77	44	33	0.429	0.127	88.0%	89.2%	0.546
2-year-old	53	31	22	0.415	0.136	79.5%	81.4%	0.548
adult—focal^c^				0.201^c^				
adult—census^d^				0.248^d^				

^a^ Binomial test, one-tailed.

^b^ Fisher exact test, one-tailed.

^c^ Based on six yearly assessments once a year in January for up to four focal groups (22 group values in total).

^d^ Based on six censuses conducted of up to six mixed-sex groups per census. ASRs are not independent (details in §2) and do not consider males outside of mixed-sex groups.

## 4. Discussion

The BSR in the Phayre’s leaf monkeys studied here was biased towards females, the dispersing sex, as predicted by LRC. Alternatively, or in addition, the BSR may be biased towards the less expensive sex or the sex with the higher mortality [17,46]. Although there is some evidence in non-human primates for this type of adjustment [47,48], in our population, interbirth intervals after male and female infants were similar (i.e. similar investment; C Borries & A Koenig 2024, unpublished data) and survival to 2 years of age was the same (see also below), leading us to suggest that the dispersal pattern and LRC govern BSR in our study system.

With 0.404, the BSR fell at the lower end for primate species with female-biased dispersal studied thus far (mean = 0.446, range = 0.380–0.499, *n* = 8; [21] (JB Silk 2023, personal communication) mean = 0.430, range = 0.410–0.450, *n* = 2, calculated from appendix in [49]). Ours is the only value for a wild population with female-biased dispersal. At present, it is unclear, whether data from captivity can be used to examine sex ratios in an evolutionary framework [21,50]. On the one hand, consistent biases across populations and species may indicate species-specific patterns relating to natural selection on genetic aspects, and captive populations would provide suitable data for comparisons [21]. On the other hand, if sex ratios can be adjusted facultatively as examples from birds and other animals have shown [13,51], captive populations may be unsuitable, because, among others, they lack selective pressures such as predation and competition for food may be low. To solve this issue, BSR data from multiple groups with known population densities, dispersal patterns, competitive regimes, etc., and crucially, known costs and benefits of sex allocation are needed [12,13].

Our study confirmed the impact of sample size on the outcome of a binary variable such as BSR. It has been suggested that the minimum sample size should be at least 100. Some of the existing BSR analyses will have to be re-evaluated considering potential sample size effects. For example, the study by Faust & Thompson [49] on BSR in captive mammals was based on 66 species but the sample size exceeded 100 births for only 46 species. Of these 46 species, the dispersal bias was considered for only 11 species, a sample size that does not allow for firm conclusions. Even very recent work may not consider sample size effects. For example, a study on captive langurs examining BSR in relation to maternal age was based on sample sizes of 1–34 births per age class [52]. Besides a sufficient sample size, again, more data for multiple groups of different sizes and for several years and different birth cohorts should be collected for wild populations.

The female-biased BSR in our study population was very similar in 2-year-olds, indicating that sex differential mortality does not seem to exist in this age class. Similarly, no changes in the sex ratio in older immatures were reported for galagoes and Nepal grey langurs [10,37], while the sex ratio bias reversed in spider monkeys [36]. These contrasting results emphasize that more data of sufficient quality need to be available before this sex ratio aspect can be analysed.

The absence of sex differential mortality in our study is consistent with the identical growth rates determined for immatures during the first 3 years of life [44]. Likewise, both sexes are of similar age when they become adults (females = 5.3 years, males = 5.1 years, cf. methods), suggesting that male Phayre’s leaf monkeys do not face a higher risk of starvation than females, as has been proposed for primates in general [53]. Still, adult males in our study species are somewhat heavier than adult females (male/female body mass ratio = 1.249; calculated based on Smith & Junger [54]), because they grow for longer [44]. Thus, they could require more food and be stronger food competitors. However, adult females face extra energetic costs during gestation and lactation [55]. Based on energy intake data considering feeding rates, bite size and plant nutrient content (methods detailed in [44,56]), it appears that in our population, these different energy requirements cancel each other out. Adult males even had a slightly lower mean energy intake rate than adult females [44].

Interestingly, the female bias in ASR in mixed-sex groups was even stronger than in 2-year-olds. Unfortunately, we know very little about juvenile and adult mortality in our study population. Most individuals simply disappeared and dead bodies were rarely recovered. Based on the ASR in mixed-sex groups, it appears that more males than females die from 2 years of age onward. We do not know if this happened mainly in older immature or in adult males. Regarding the risks of dispersal [57], females are likely to disperse further than males. However, females usually switched from one mixed-sex group to the next during group encounters (C Borries *et al*. 2024, unpublished data), while males may spend time as solitaries or with a few other males [39]. This could translate into a higher mortality risk for males outside of mixed-sex groups given the intact predator community and several confirmed cases of predation by felids at our study site [58–60]. In addition, resident males seem to compete fiercely among each other (eye-witnessed accounts in [39]) as suggested by their significantly higher rate of injuries compared to females [61]. Males also have a higher variance in reproductive success and might thus take more risks. For example, one adult male in the population sired 18 infants and may have sired five additional ones for which no samples were available [40]. In comparison, the maximum number of births documented for a female was five, which happened twice. Taken together, males are likely exposed to higher risks and might take higher risks than females, thus lowering their chances of survival. While speculative, this might explain the stronger female-biased sex ratio in adults.

Overall, our study suggests LRC to occur in wild Phayre’s leaf monkeys corresponding to a female-biased BSR. The specific characteristics of the local resources contested still need to be examined. Our study re-emphasizes the importance of a large sample size (at least 100 births) for BSR [21] and suggests to also consider the potential long-term effects of BSR biases by comparing them to ASR.

## Data Availability

All data used are included in the tables provided in the manuscript.
